# Association between Alzheimer’s disease-related genes and neurodegenerative blood-based biomarkers in Indian adults

**DOI:** 10.3389/fnagi.2026.1832155

**Published:** 2026-08-19

**Authors:** Hasan Abu-Amara, Wei Zhao, Zheng Li, Yuk Yee Leung, Gerard D. Schellenberg, Li-San Wang, Eileen M. Crimmins, Bharat Thyagarajan, Masroor Anwar, Perry Hu, Sharmistha Dey, Aparajit B. Dey, Xiang Zhou, Kumarasamy Thangaraj, Jinkook Lee, Sharon L. R. Kardia, Jennifer A. Smith

**Affiliations:** 1 Department of Epidemiology, School of Public Health, University of Michigan, Ann Arbor, MI, United States; 2 Survey Research Center, Institute for Social Research, University of Michigan, Ann Arbor, MI, United States; 3 Department of Biostatistics, School of Public Health, University of Michigan, Ann Arbor, MI, United States; 4 Department of Pathology and Laboratory Medicine, Perelman School of Medicine, University of Pennsylvania, Penn Neurodegeneration Genomics Center, Philadelphia, PA, United States; 5 Davis School of Gerontology, University of Southern California, Los Angeles, CA, United States; 6 Department of Laboratory Medicine and Pathology, University of Minnesota, Minneapolis, MN, United States; 7 Department of Biophysics, All India Institute of Medical Sciences, New Delhi, India; 8 Department of Medicine/Geriatrics, Geffen School of Medicine, University of California, Los Angeles, Los Angeles, CA, United States; 9 Department of Geriatric Medicine, Artemis Hospital, Gurugram, India; 10 Evolutionary and Medical Genetics Laboratory, Centre for Cellular and Molecular Biology, Hyderabad, India; 11 Department of Economics, University of Southern California, Los Angeles, CA, United States

**Keywords:** Alzheimer’s disease, cognitive function, gene-based analysis, gene-by-age interaction, gene-by-sex interaction, genetics, neurodegenerative biomarkers, rare variants

## Abstract

**Background:**

Alzheimer’s disease (AD) and related dementias are a growing health and economic burden in India. Blood levels of amyloid beta (Ab), tau, and other proteins marking neuronal injury are biomarkers of AD risk and are potentially important for AD prevention.

**Methods:**

In 2,224 participants from the Harmonized Diagnostic Assessment of Dementia for the Longitudinal Aging Study in India (LASI-DAD), we performed gene-based analyses on (1) missense/loss-of-function (LoF) single-nucleotide variants (SNVs) and (2) brain-specific promoter/enhancer SNVs across 84 genes selected from AD GWAS and 25 gene-biomarker pairs selected from biomarker GWAS. We used variant-Set Test for Association using Annotation infoRmation (STAAR) across 7 neurodegenerative biomarkers measured in blood: Ab40, Ab42, Ab42/Ab40, total tau (tTau), tau phosphorylated at threonine 181 (pTau), glial fibrillary acidic protein (GFAP), and neurofilament light chain (NfL). We adjusted for age, sex, and genetic ancestry, with random intercepts for genetic relatedness and biomarker plate. Analyses incorporated weighted annotation scores (e.g., deleteriousness). Significant results (FDR-*q* < 0.1) were followed up with single variant analysis. Genes were also assessed for sex- and age-interactions using iSKAT.

**Results:**

Missense/LoF variants in 6 AD-associated genes (*ECHDC3*, *CLU*, *APOE*, *EPHA1*, *ICA1L*, *CASS4*) and 1 biomarker-associated gene (*APOE*) were associated with Ab40, Ab42/Ab40, pTau, and/or GFAP (FDR *q* < 0.1), with the most significant SNVs tending to be rare/low-frequency (MAF ≤ 0.05) and potentially deleterious. Promoter/enhancer variants in 1 AD-associated gene (*CCDC6*) were associated with Ab42/Ab40, with the index SNV being a potentially deleterious common variant (MAF = 0.27). Several associated variants appeared to have higher frequency in India compared to other global populations. Missense/LoF SNVs in two AD genes (*EPHA1*, *MS4A6A*) had sex-specific effects on Ab42 and/or tTau, and promoter/enhancer SNVs in four AD genes (*DGKQ*, *MS4A6A*, *MS4A4A*, *JAZF1*) had sex-specific effects on tTau and/or pTau. Missense variants in 12 AD genes and promoter/enhancer variants in two AD genes showed interaction with age on at least one biomarker, primarily GFAP and NfL with genetic effects tending to be stronger at older ages.

**Conclusion:**

Rare and common variants in AD- and neurodegenerative biomarker-associated genes with increased frequency in India compared to other populations may impact blood levels of neurodegenerative biomarkers in South Asians.

## Introduction

Dementia is a group of neurological disorders resulting from decreased cognitive function and memory. The most prevalent form of dementia is Alzheimer’s Disease (AD), which is caused by accumulation of amyloid β plaques and neurofibrillary tangles in the brain, leading to neurological degeneration. The number of people living with dementia globally was estimated to be 57 million in 2019 and was projected to grow to 153 million by 2050 (Nichols et al., 2022). Much of this burden will be borne by lower- and middle-income countries (Wimo et al., 2023). Care for individuals with dementia is often financially costly as well as physically and emotionally burdensome for caregivers.

Recently, neurodegenerative biomarkers sampled from cerebral spinal fluid and blood, such as amyloid β 42 and tau phosphorylated at threonine 181 (Jack et al., 2024), were recommended for use in AD diagnosis in conjunction with classical AD symptoms (Jack et al., 2018). Genetic studies of neurodegenerative biomarkers have begun to identify associated genetic loci (Damotte et al., 2021; Jansen et al., 2022; Sarnowski et al., 2022), which may improve knowledge of AD etiology as these biomarkers are more proximal to AD pathology than clinical symptoms (Jansen et al., 2022). Additionally, the focus on neurodegenerative biomarkers allows finer resolution on AD-related biological processes compared to a macro-level phenotype such as cognitive function or AD case status. The use of neurodegenerative biomarkers as an endophenotype increases statistical power to detect potential novel genetic associations with AD as well. To date, most genome-wide association studies (GWAS) of neurodegenerative biomarkers have been conducted in European Ancestry (EA) cohorts (Damotte et al., 2021; Jansen et al., 2022; Sarnowski et al., 2022), so there is a great need to expand this line of research into non-European populations.

India is currently the most populous country in the world and has a dementia prevalence of approximately 7.4% among individuals aged 60 and over (Lee J. et al., 2023). However, few genetic studies have been conducted in India. Because of this, it is unclear whether genetic loci identified in primarily EA GWAS would be associated with AD or its neurodegenerative biomarkers in Southeast Asians living in India. Further, there may be genetic variants more frequent in South Asians that are associated with neurodegenerative biomarkers that have yet to be discovered. Additionally, examination of genetic associations with these biomarkers across demographic characteristics, such as sex and age, is warranted. For example, among *APOE*ε4 carriers, women may have larger tau loads compared to men (Boccalini et al., 2025), and amyloidosis associated with elderly age may be more frequent in women as well (Jack et al., 2014).

In this study, we examined whether genes previously associated with AD and neurodegenerative biomarkers in primarily EA GWAS were associated with seven neurodegenerative biomarkers in 2,224 participants from the nationally representative Harmonized Diagnostic Assessment of Dementia for Longitudinal Aging Study of India (LASI-DAD). We then assessed whether the detected associations appear to be driven by variants more frequent in South Asians compared to other global populations. We also evaluated whether the genetic effects on the biomarkers differed by sex or age. We focused on single-nucleotide variants (SNVs) that were missense or loss-of-function (LoF) variants, and on those that were in brain-specific promoter/enhancer regions. We used gene-based analysis to maximize power by aggregating genetic signal across rare variants within a single gene, and because gene-centric analysis accommodates differences in linkage disequilibrium (LD) structure across ancestries. Identifying SNVs associated with neurodegenerative biomarkers in South Asians may ultimately improve risk stratification and AD prevention.

## Materials and methods

### Study population

LASI-DAD is an ancillary study of the Longitudinal Aging Study of India (LASI) that investigates dementia risk factors in a nationally representative sample of Indians at least 45 years old (Perianayagam et al., 2022). LASI-DAD includes 4,096 LASI participants at least 60 years old selected via two-stage stratified random sampling from 18 administrative states and union territories in India to ensure a representative cognitive impairment distribution similar to the population distribution (Lee and Dey, 2020; Lee et al., 2020). Participants underwent neurocognitive testing, participated in interviews, and provided blood samples for whole genome sequencing (WGS). The analytic sample includes a total of 2,224 participants with at least one neurodegenerative biomarker measure and genotype data available.

### Neurodegenerative biomarkers

Neurodegenerative biomarkers were measured from plasma specimens using a single molecule array (Simoa) based Quanterix HD-X*™* instrument (Quanterix Corporation, Billerica, MA, United States). Assays for the biomarkers were performed at the Department of Biophysics at the All India Institute of Medical Sciences (AIIMS), New Delhi (Khobragade et al., 2024). Six neurodegenerative biomarkers were assayed: amyloid β 40 (Ab40), amyloid β 42 (Ab42), total tau protein (tTau), tau phosphorylated at threonine 181 (pTau), glial fibrillary acidic protein (GFAP), and neurofilament light chain (NfL). The Ab42/Ab40 ratio (Ab42/Ab40) was also calculated and analyzed as a 7th biomarker. After removal of three outlying values for NfL/GFAP, the intra-assay coefficients of variation ranged from 5.66 to 6.99% (Kim et al., 2025). Neurodegenerative biomarkers were log_10_ transformed, converted to z-scores, and Winsorized to five standard deviations, with one to 13 outliers Winsorized per biomarker. The respective plates for each neurodegenerative biomarker were used as random intercepts in statistical models to adjust for potential plate effects. For five participants, Ab40 and Ab42 were measured on different plates; in these cases, the Ab40 plate was used to adjust for plate effects in analyses of Ab42/Ab40.

### Whole-genome sequencing data

WGS was performed at an average depth of 30x by MedGenome, Inc. (Bangalore, India) with blood samples from 2,762 LASI-DAD participants. Genotype calling and quality control was performed by the Genome Center for Alzheimer’s Disease (GCAD) (Leung et al., 2019). Additional details can be found elsewhere (Abu-Amara et al., 2025). A total of 66,204,161 autosomal bi-allelic single nucleotide variants were retained for analysis.

### Principal component analysis and genetic relatedness matrix

Genetic principal components (PCs) and the genetic relatedness matrix (GRM) were estimated in GENESIS (version 2.26.0) (Gogarten et al., 2019; Abu-Amara et al., 2025). PCs were estimated among a set of unrelated individuals (kinship cutoff = 0.088) to obtain robust variant weights, then were used for PC projection in the rest of the sample. The GRM was estimated in unrelated samples with “PCrelate” by adjusting the top two PCs to avoid potential confounding from population structure. The first PC was strongly correlated with Ancestral South Indian ancestry (Pearson correlation coefficient *r* = −0.98), and the first 10 PCs cumulatively explained 48% of the variance in global ancestry.

### Gene selection

We selected genes previously associated with AD and neurogenerative biomarkers from large GWAS. Briefly, we included a total of 84 genes from two GWAS that demonstrated genome-wide significant association with AD (Wightman et al., 2021) or AD and related dementias (Bellenguez et al., 2022). In total, we selected 73 genes from Bellenguez et al. (2022) and 45 genes from Wightman et al. (2021) or with 36 genes overlapping between the two GWAS (Supplementary Table S1). Participants from both GWAS were EA. AD-associated gene selection was previously described (Abu-Amara et al., 2025). Next, we selected 22 genes that demonstrated association at a genome-wide level with neurodegenerative biomarkers (Ab42, Ab42/Ab40, tTau, and pTau) from three GWAS (Damotte et al., 2021; Jansen et al., 2022; Sarnowski et al., 2022; Supplementary Tables S2, S3). No GWAS on GFAP or NfL were available. We selected genes that had nearby variants associated with Ab42 or pTau (*p* < 5 × 10^–8^) in a stage 3 meta-analysis of genotype chip data (Jansen et al., 2022), were associated with Ab42 and/or Ab42/Ab40 in gene-based tests (*p* < 2.76 × 10^–6^) from genotype chip data (Damotte et al., 2021), or were associated with tTau in gene-based tests from whole exome sequence data (Sarnowski et al., 2022). We analyzed the genes associated with neuorodegenerative biomarkers in EA GWAS only with the biomarkers they were previously associated with, resulting in 25 unique neurodegenerative gene-biomarker pairs from the 22 selected genes.

Gene boundaries were defined using start and stop sites in GENCODE v47 (Frankish et al., 2023). We selected all missense/LoF variants within gene boundaries, and all brain-specific promoter and enhancer variants within ± 5 kb and ± 20 kb of the transcription start sites, respectively. Genes were only retained for analysis if they had at least two missense/LoF or brain-specific promoter/enhancer SNVs with minor allele count (MAC) ≥ 5.

### Missense/LoF and promoter/enhancer variants

We used Variant Effect Predictor (VEP) (McLaren et al., 2016) and LOFTEE (Karczewski et al., 2020) with GENCODE as a transcript reference to identify SNVs with missense and LoF as their functional consequence. We then used the WGS Annotator (WGSA) v0.95 pipeline (Liu et al., 2015) to retrieve H3K4me3 annotations for brain tissues (E067-E074, E081, E082) and enhancer annotations provided by EnhancerFinder for the brain from the ENCODE database. Detailed methods are described elsewhere (Abu-Amara et al., 2025).

### Annotation selection

We incorporated several annotation scores similar to Lee et al. (2022) for the gene-based analysis, which measured deleteriousness, impact on the protein, and evolutionary conservation of the SNV. We selected CADD_raw_rankscore (Rentzsch et al., 2019), GERP_RS_rankscore (Davydov et al., 2010), Eigen.Phred (Ionita-Laza et al., 2016), and fathmm.MKL_coding_rankscore (Shihab et al., 2015) for the missense/LoF analysis; and CADD_raw_rankscore (Rentzsch et al., 2019), GERP_RS_rankscore (Davydov et al., 2010), Eigen.PC.phred (Ionita-Laza et al., 2016), GenoCanyon_rankscore (Lu et al., 2015), and fathmm.MKL_noncoding_rankscore (Shihab et al., 2015) for the brain-specific promoter/enhancer analysis. Additional details can be found elsewhere (Abu-Amara et al., 2025).

### Statistical methods

All analyses were conducted in R (v4.5.1). VCF files were converted to GDS files using the SeqArray package (Zheng et al., 2017). We tested the two gene sets separately for associations with each neurodegenerative biomarker. AD-associated genes were tested with all seven biomarkers, while neurodegenerative biomarker-associated genes were tested only with the biomarkers each gene was previously associated with in previous GWAS (Supplementary Table S2).

#### STAAR gene-based analysis

Gene-based analysis for associations with neurodegenerative biomarkers were assessed with the variant-Set Test for Association using Annotation infoRmation (STAAR) v0.9.6.1 (Li et al., 2020) on missense/LoF SNVs and brain-specific promoter/enhancer SNVs separately. Linear mixed models were used to test each set of genes for associations with the biomarkers, adjusting for age, sex, and the first 10 principal components of global ancestry with random intercepts for biomarker plate and adjusting for relatedness using a GRM. STAAR generated two separate omnibus *p*-values for analyses with (STAAR-O) and without (ACAT-O) annotation scores. Each omnibus *p*-value was calculated from the SKAT, Burden, and ACAT-V tests using Beta(1, 1) and Beta(1, 25) weighting with the minor allele frequency of the SNVs. Only genes with at least two SNVs with a complete set of annotations and MAC ≥ 5 were analyzed. Since this study is exploratory, we used a relatively liberal threshold to declare significance. Genes were considered associated if they had a Benjamini-Hochberg FDR *q* < 0.1 in LASI-DAD.

For genes significantly associated with a neurodegenerative biomarker, we performed a single-variant follow-up analysis to better characterize the variants driving the gene-based associations. Beta estimates for each SNV within the gene were calculated in GENESIS using the same model tested in STAAR, and those with the smallest *p*-values in each gene were designated as sentinel SNVs for that association. To determine if the identified SNVs are potentially higher frequency in India compared to other populations, we compared the frequency of the minor allele in LASI-DAD to those of the non-Finnish European (NFE), East Asian (EAS), African (AFR), Middle Eastern (MID), and South Asian (SAS) samples in gnomAD v4.1.0 (Chen et al., 2022). We also queried the GTEx Portal (accessed 05/30/2026) to examine whether any of the sentinel SNVs were associated with differential gene expression in brain tissue from a sample of 943 individuals of primarily European ancestry (Lonsdale et al., 2013). For each identified gene, plots of the SNV-level associations with the associated biomarker were produced with locuszoomr v0.3.8 (Lewis and Wang, 2025) and LD information calculated as the unphased r^2^ with PLINK 2.0 (Purcell et al., 2007) among the entire LASI-DAD sample with genomic data available. SNV recombination rates were calculated as an average over the 1,000 Genomes GRCh38 reference panels from the University of California, Santa Cruz, lifted over from hg19. Gene tracks were retrieved from Ensembl 113 (Harrison et al., 2024) via AnnotationHub v3.16.1 (Morgan and Shepherd, 2025).

As a sensitivity analysis to examine the effect of study site, for each gene significantly associated with a neurodegenerative biomarker, we further adjusted for state/territory as a categorical variable and compared the *p*-value to that of the primary association.

#### Gene-by-sex and gene-by-age interaction analysis

Gene-by-sex and gene-by-age interactions were tested using the interaction sequence kernel association test (iSKAT) (Lin et al., 2015). We tested missense/LoF and brain-specific promoter/enhancer SNVs with a minor allele frequency (MAF) > 0 and complete annotation score information in each gene separately. We tested interactions with both sex and age, in separate models, and used Beta(1, 25) variant weights. iSKAT output includes an overall *p*-value and ρ, a parameter that indicates whether a burden-like test (ρ = 1) or a sequence kernel association test-like (SKAT, ρ = 0) is weighted more heavily in the optimization. The burden test assumes all variants have similar effect magnitudes and directions, while the SKAT test allows opposite effect directions and varying effect sizes. Because iSKAT cannot use a linear mixed model, we first extracted residuals from an intercept-only linear mixed model with plate and GRM as random intercepts and then added the sample mean of the biomarker to the residuals. We adjusted for age, sex, and the first 10 principal components of global ancestry. SNVs with missingness ≤ 20% and MAF > 0% in the analytic sample for a specific biomarker were tested. Additionally, the gene-by-sex interaction analysis included only SNVs with MAC ≥ 5 in both sexes, and the gene-by-age interaction analyses included only SNVs with MAC ≥ 5. Genes were considered associated if they had a Benjamini-Hochberg FDR *q* < 0.1.

For genes with a significant sex or age interaction, we performed a single-variant follow-up analysis with the SNV that had the smallest interaction *p*-value in iSKAT. Using the model and residuals described above, GENESIS v2.38.0 (Gogarten et al., 2019) was used to generate male- and female-specific effects (for sex interactions) or a main and interaction term estimate (for age interactions). Sex-specific estimates were obtained by fitting the null model twice, using either males or females as the reference group. These effects were tested using a linear regression model with the SNV as a main effect, its interaction with sex or age, and adjusting for all covariates above. To visualize interactions with age, we provide predicted standardized biomarker plots by age and minor allele dosage for the sentinel SNV of each detected age-variant interaction, including 95% confidence bands for the mean, plotted using the emmeans R package (Lenth, 2023).

## Results

LASI-DAD participants with neurodegenerative biomarker and genetic data had a mean age of 69.58 years (SD = 7.38) and was majority female (*N* = 1,195; 54%). The number of participants in the analytic sample varied with each biomarker, ranging from 1,824 for Ab42/Ab40 to 2,192 for tTau (Table 1). Correlations between biomarkers varied widely from 0.82 to −0.28 (Supplementary Figure S1). GFAP and NfL had moderate correlations with pTau, tTau, Ab42, and Ab40.

**TABLE 1:** Characteristics of the LASI-DAD analytic sample (*N* = 2,224).

Variable name	N	Minimum	Maximum	Mean or N	SD or %
Sex (female)	2,224	-	−	1,381	54%
Age	2,224	60	102	69.58	7.38
Neurodegenerative biomarkers*					
Amyloid beta 40 (Ab40)	2,106	0.16	293.03	52.04	38.22
Amyloid beta 42 (Ab42)	1,829	0.04	87.39	3.41	2.89
Ab42/Ab40	1,824	0.01	1.70	0.06	0.05
Total Tau (tTau)	2,192	0.00	143.98	2.81	5.65
Phosphorylated Tau-181 (pTau)	2,189	0.59	782.03	41.61	32.15
Glial fibrillary acidic protein (GFAP)	2,174	0.01	1267.56	151.77	109.61
Neurofilament light chain (NfL)	2,124	0.00	1165.52	43.94	59.84

*Biomarkers were Log_10_ Z-Score transformed and Winsorized to 5 SD prior to analysis.

The distribution of missense/LoF and brain-specific SNVs analyzed with tTau, which has the largest analytic sample size, is presented in Supplementary Table S4. The median number of missense/LoF SNVs analyzed per gene was about 6 for AD-associated genes and 3 for neurodegenerative biomarker-associated genes, and the median number of promoter/enhancer SNVs analyzed per gene was about 22 for AD-associated genes and 18.5 for neurodegenerative biomarker-associated genes. Median functional annotation weights tended to be highest in missense/LoF SNVs compared to promoter/enhancer SNVs (Supplementary Tables S5, S6).

### Gene-based analysis

#### Missense/loss-of-function (LoF) analysis

Of the 84 AD-associated genes, 59–61 genes met the criteria for analysis across the different neurodegenerative biomarkers. All 25 neurodegenerative gene-biomarker pairs selected from previous literature met the criteria for analysis.

Without annotation weights, 5 AD-associated genes (*CLU, APOE, EPHA1, ICA1L, CASS4*) were associated with at least one neurodegenerative biomarker after correction for multiple testing (FDR *q* < 0.1, Table 2). Specifically, *CLU* (*q* = 0.096) and *APOE* (*q* = 0.096) were associated with Ab42/Ab40, *ICA1L* (*q* = 0.089) and *CASS4* (*q* = 0.089) were associated with pTau, and *EPHA1* was associated with GFAP (*q* = 0.0023). Of the neurodegenerative biomarker-associated genes, only *APOE* (*q* = 0.0040) had an association with a biomarker in LASI-DAD (Ab42/Ab40, Table 2). After incorporating annotation weights, only two AD-associated genes (*ECHDC3*, *EPHA1*) were associated with Ab40 (*q* = 0.074) and GFAP (*q* = 0.00058), respectively. The *APOE* association with Ab42/Ab40 persisted after incorporating annotation weights (*q* = 0.0040). Adjusting for state/territory did not change the *p*-values appreciably (Supplementary Table S7).

**TABLE 2:** Previously identified genes that were associated with neurodegenerative biomarkers (FDR *q* < 0.1) in the LASI-DAD gene-based analysis.

Neurodegenerative biomarker	Gene	# of SNVs analyzed	cMAC	*P*-value (with annotation weights)	*P*-value (without annotation weights)	FDR *q*-value (with annotation weights)	FDR *q*-value (without annotation weights)	Smallest *p*-value (SKAT, ACAT-V, or burden test)	Weight of most significant test
Missense/loss-of-function SNVs in AD-associated genes									
Ab40	*ECHDC3*	4	798	**0.0012**	0.0017	**0.074**	0.10	ACAT-V	Beta(1, 25)
Ab42/Ab40	*CLU*	3	26	0.0039	**0.0034**	0.11	**0.096**	ACAT-V	Beta(1, 1)
Ab42/Ab40	*APOE*	2	584	0.0020	**0.0020**	0.11	**0.096**	SKAT	Beta(1, 1)
pTau	*ICA1L*	3	68	0.0038	**0.0030**	0.12	**0.089**	Burden	Beta(1, 25)
pTau	*CASS4*	9	393	0.0027	**0.0030**	0.12	**0.089**	ACAT-V	Beta(1, 25)
GFAP	*EPHA1*	11	755	**9.61E-06**	**3.76E-05**	**0.00058**	**0.0023**	ACAT-V	Beta(1, 25)
Brain-specific promoter/enhancer SNVs in AD-associated genes									
Ab42/Ab40	*CCDC6*	27	9,937	**0.0011**	**0.0012**	**0.084**	**0.090**	Burden	Beta(1, 1)
Missense/loss-of-function SNVs in neurodegenerative biomarker-associated genes									
Ab42/Ab40	*APOE*	2	584	**0.0020**	**0.0020**	**0.0040**	**0.0040**	SKAT	Beta(1, 1)
Brain-specific promoter/enhancer SNVs in neurodegenerative biomarker-associated genes									
Ab42/Ab40	*APOE*	17	6,019	0.060	**0.055**	0.12	**0.082**	ACAT-V	Beta(1, 1)
Ab42/Ab40	*APOC1*	19	5,686	0.080	**0.047**	0.12	**0.082**	ACAT-V	Beta(1, 1)

Bolded values have FDR *q* < 0.1. Ab40, amyloid β 40; Ab42, amyloid β 42; Ab42/Ab40, Ab42 to Ab40 ratio; AD, Alzheimer’s disease; cMAC, cumulative minor allele count; FDR, false discovery rate; GFAP, glial fibrillary acidic protein; pTau, tau phosphorylated at threonine 181; SNV, single nucleotide variant.

**FIGURE 1:**
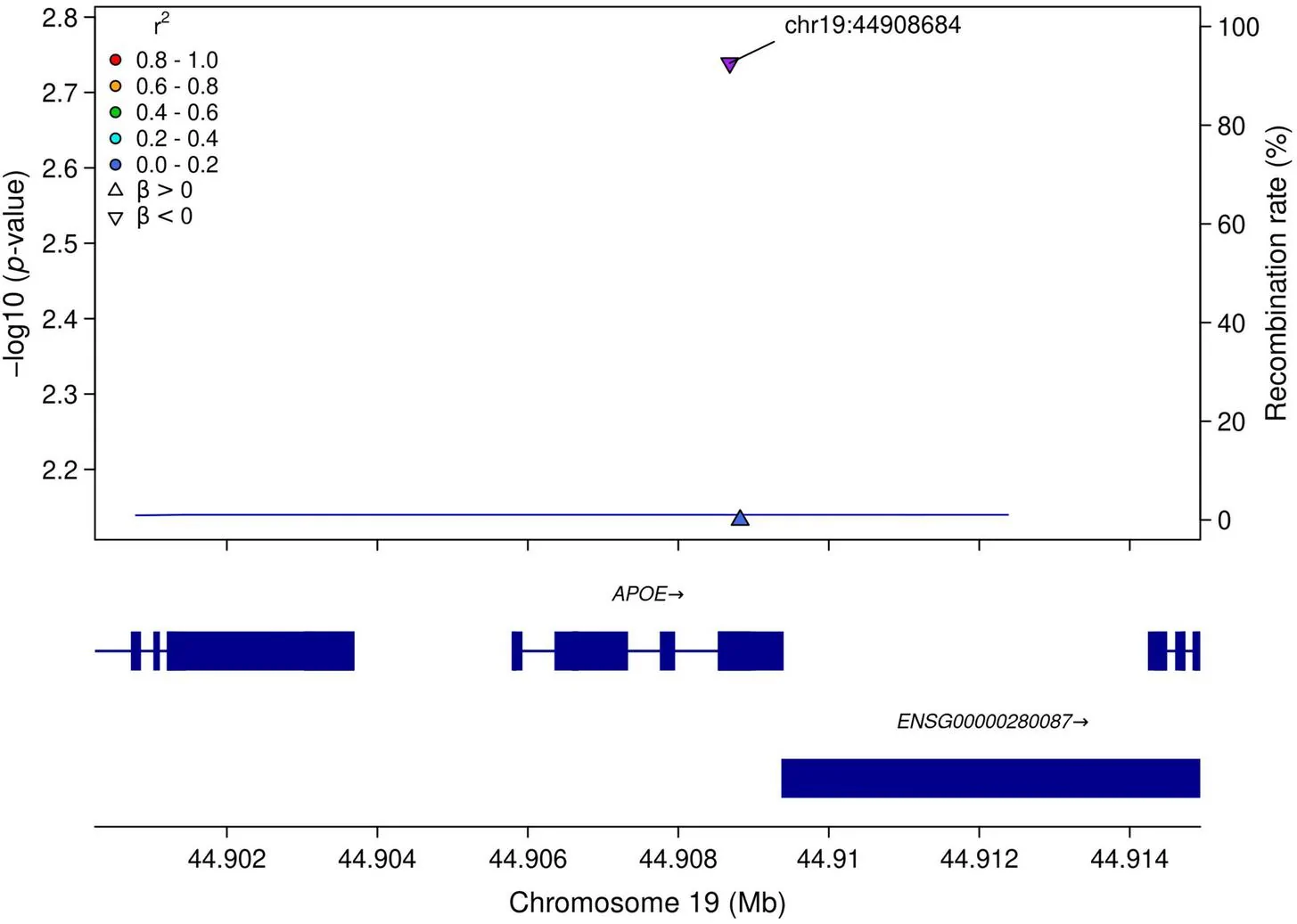
Plot of missense/loss-of-function SNVs with MAC ≥ 5 in *APOE* for Ab42/Ab40. Only ε4 (rs429358) and ε2 (rs7412) had MAC ≥ 5 in *APOE.* Left *Y*-axis: −log10 (*p*-value) of the association between the SNV and Ab42/Ab40, adjusting for age, sex, and the first 10 principal components of genetic ancestry, and accounting for relatedness and plate as random effects; Right *Y*-axis: SNV recombination rate averaged over all 1,000 genomes GRCh38 reference panels, lifted over from hg19, queried from the University of California, Santa Cruz; *X*-axis: chromosomal location and gene regions. LD information is calculated as the unphased *r*^2^ from the LASI-DAD analytic sample. The LD *r*^2^ color code indicates the degree of linkage between the SNV and the index (most strongly associated) SNV, rs429358 (purple triangle). No annotation or MAF weighting was used to calculate the *p*-value. The effect direction for SNVs with *p* < 0.05 is indicated by triangle direction (up indicates positive effect, down indicates negative). The minor allele of the most significant SNV (rs429358, ε4) was associated with lower Ab42/Ab40, which is linked to higher AD risk.

Table 3 shows the effect estimates and *p*-values for the SNVs in each gene that were most strongly associated with the biomarkers (sentinel SNVs), and Supplementary File S1 lists all marginally associated variants (*p* < 0.05) within each gene that reached significance (FDR *q* < 0.1). The SNV with the smallest *p*-value in *APOE* was rs429358, which marks the ε4 allele (Ab42/Ab40 β = −0.15, *p* = 0.0018, Figure 1 and Supplementary Figures S2–S6). As expected based on the higher risk of AD for ε4 carriers, the minor allele was negatively associated with Ab42/Ab40 (Vogelgsang et al., 2019). Sentinel SNVs in all genes except *CLU* tended to have high CADD Phred scores ( > 10, indicating that they are within the top 10% of all deleterious variants), and tended to be more prevalent in LASI-DAD compared to other global populations. They also tended to be rare or low-frequency variants, with MAFs ranging from 0.03 to 5.01%, with only rs429358 (*APOE*ε4) being common (MAF = 11%). Some SNVs (rs141275729, rs769966727, rs371557794) had higher MAFs in the analytic sample compared to NFE, EAS, AFR, and MID populations, potentially indicating higher frequency in India. Only one sentinel SNV was associated with gene expression in brain tissue in GTEx (rs35986488 in *ECHDC3*). The minor alleles of the sentinel SNVs were associated with biomarker levels indicative of increased risk of AD, except for those in *EPHA1*, *CASS4*, and *CLU*.

**TABLE 3:** Sentinel SNVs in genes that were associated with a neurodegenerative biomarker in LASI-DAD.

Neuro- degenerative biomarker	Gene	rsID	ID	Minor allele	MAF (analytic)	AF (EAS)	AF (AFR)	AF (MID)	AF (NFE)	AF (SAS)	MAC	β	*P*-value	Consequence	CADD Phred	eQTL tissue type
Missense/loss-of-function SNVs in AD-associated genes																
Ab40	*ECHDC3*	rs35986488	10:11755 501:G:A	A	0.050	0.0021	0.045	0.075	0.087	0.058	211	−0.23	0.00033	Missense	31	Amygdala, anterior cingulate cortex, caudate, cortex, frontal cortex, hippocampus, hypothalamus, nucleus accumbens. putamen, substantia nigra
Ab42/Ab40	*APOE*	rs429358	19:44908 684:T:C	C	0.11	0.098	0.22	0.068	0.14	0.10	403	−0.15	0.0018	Missense	16.65	None
Ab42/Ab40	*CLU*	rs371557794	8:27605 044:G:C	C	0.0041	0	0	0	0	0.0037	15	0.76	0.0017	Missense	8.37	N/A
pTau	*CASS4*	rs769966727	20:56445 973:C:A	A	0.0030	0	0	0	0	0.00041	13	−1.07	7.60E-05	Missense	12.32	N/A
pTau	*ICA1L*	rs144482724	2:20282 1479:T:C	C	0.0050	0	0.00094	0.017	0.0039	0.0039	22	0.53	0.0085	Missense	17.95	N/A
GFAP	*EPHA1*	rs141275729	7:143399 973:G:C	C	0.0016	0	0	0	2.94E−05	0.00083	7	−1.55	8.36E-06	Missense	26.3	None
Brain-specific promoter/enhancer SNVs in AD-associated genes																
Ab42/Ab40	*CCDC6*	rs1171833	10:5990 8809:G:A	G	0.27	0.10	0.29	0.32	0.28	0.27	982	−0.12	0.00065	Promoter	19.57	None
Missense/loss-of-function SNVs in neurodegenerative biomarker-associated genes																
Ab42/Ab40	*APOE*	rs429358	19:4490 8684:T:C	C	0.11	0.098	0.22	0.068	0.14	0.10	403	−0.15	0.0018	Missense	16.65	None
Brain-specific promoter/enhancer SNVs in neurodegenerative biomarker-associated genes																
Ab42/Ab40	*APOE*	rs429358	19:44908 684:T:C	C	0.11	0.098	0.22	0.068	0.14	0.10	403	−0.15	0.0018	Missense	16.65	None
Ab42/Ab40	*APOC1*	rs7256200	19:4491 2678:G:T	T	0.07	0.096	0.19	0.037	0.11	0.065	258	−0.20	9.03 × 10^–4^	Promoter	0.20	None

Expression quantitative trait locus (eQTL) tissue type refers to the brain tissue(s) in GTEx for which the variant was an eQTL. N/A indicates that the variant was not present in GTEx. Ab40, amyloid β 40; Ab42, amyloid β 42; Ab42/Ab40, Ab42 to Ab40 ratio; AD, Alzheimer’s disease; AF, Allele Frequency; AFR, African; CADD, Combined Annotation Dependent Depletion; EAS, East Asian; eQTL, expression quantitative trait locus; GFAP, glial fibrillary acidic protein; MAC, minor allele count; MAF, minor allele frequency; MID, Middle Eastern; NFE, non-Finnish European; pTau, tau phosphorylated at threonine 181; SAS, South Asian; SNV, single nucleotide variant.

#### Brain-specific promoter/enhancer SNV analysis

**FIGURE 2:**
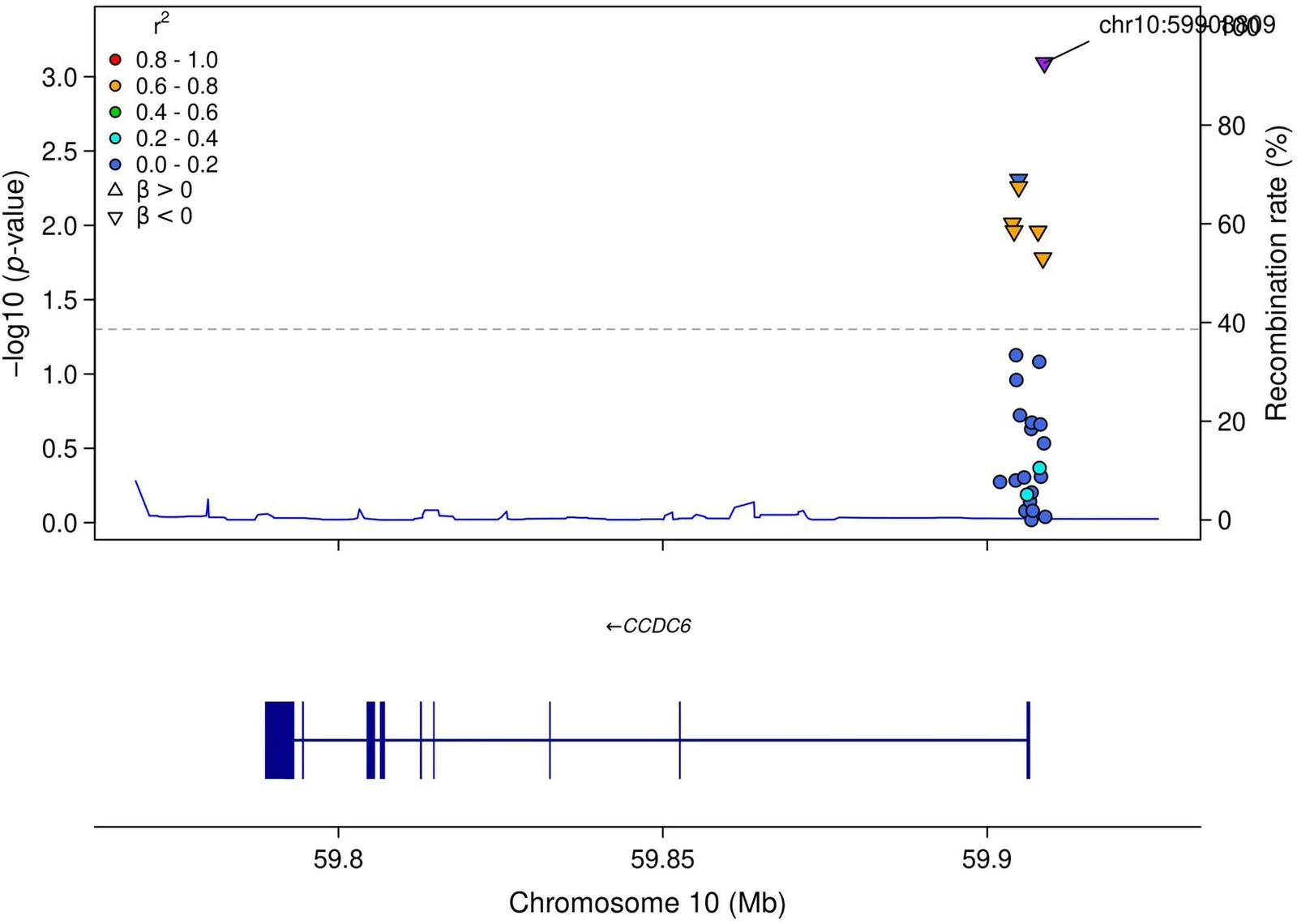
Plot of brain-specific promoter/enhancer SNVs with MAC ≥ 5 in *CCDC6* gene for Ab42/Ab40. Left *Y*-axis: −log10 (*p*-value) from association between SNV and Ab42/Ab40, adjusting for age, sex, and the first 10 principal components of genetic ancestry, and accounting for relatedness and plate as random effects; Right *Y*-axis: SNV recombination rate is averaged over all 1,000 genomes GRCh38 reference panels, lifted over from hg19, queried from the University of California, Santa Cruz; *X*-axis: chromosomal location and gene regions; LD information is calculated as the unphased *r*^2^ calculated on the LASI-DAD analytic sample; LD *r*^2^ color code indicates the degree of linkage between SNVs and the index (most strongly associated) SNV, rs1171833 (purple triangle). No annotation or MAF weighting was used to calculate *p*-values. Effect direction for SNVs with *p* < 0.05 is indicated by triangle direction (up indicates positive effect, down indicates negative). The minor alleles of all SNVs with *p* < 0.05 were associated with lower blood Ab42/Ab40, which is linked to higher AD risk.

Of the 84 AD-associated genes, 74–75 genes met the criteria for analysis across the different neurodegenerative biomarkers, and all 25 neurodegenerative gene-biomarker pairs met the criteria. Without annotation weights, one AD-associated gene (*CCDC6*, FDR *q* = 0.090) was associated with Ab42/Ab40, with the burden test showing the lowest *p*-value (Table 2). *APOE* (*q* = 0.082) and *APOC1* (*q* = 0.082) were the only neurodegenerative biomarker-associated genes associated with Ab42/Ab40 (Table 2). Results were similar, though not significant, with annotation weights incorporated. Adjusting for state/territory did not change the *p*-values appreciably (Supplementary Table S7). In *CCDC6*, the SNV with the smallest *p*-value was rs1171833 (β = -0.12, *p* = 0.00065, MAF = 0.27), which was predicted to be deleterious (CADD Phred = 19.57) and had similar MAF in NFE samples. Most variants in *CCDC6* tended to be associated with lower Ab42/Ab40 which is indicative of higher AD risk (Figure 2). As above, rs429358 (*APOE* ε4) was associated with lower Ab42/Ab40. In *APOC1*, the sentinel SNV, rs7256200, was also associated with lower Ab42/Ab40 (β = −0.20, *p* = 9.03 × 10^–4^) (Supplementary Figures S7, S8).

### Interaction between genes and sex

#### Missense/LoF SNV analysis

A total of 71–72 AD-associated genes and 23 of the neurodegenerative gene-biomarker pairs met the criteria for the analysis of gene-by-sex interactions. Two AD-associated genes (*EPHA1, MS4A6A*) had interactions with sex on Ab42 (*p* = 0.0022, FDR *q* = 0.094) and tTau (*p* = 0.0053, FDR *q* = 0.024), respectively (Table 4). No neurodegenerative-biomarker genes interacted with sex. Sentinel SNVs for the gene-sex interactions are shown in Supplementary Table S8. The variant with the smallest sex interaction *p*-value in *EPHA1*, rs6967117, was predicted to be deleterious (CADD Phred = 11.4), and each copy of the minor allele (T) was associated with lower Ab42 in females but higher Ab42 in males (β_female_ = −0.22, β_male_ = 0.30, GxE *p* = 0.00078). Similarly, the minor allele (G) of the variant with the smallest sex interaction *p*-value in *MS4A6A*, rs61742546, was associated with lower tTau in males but not females (β_male_ = −0.54, β_female_ = 0.26, GxE *p* = 0.00051) and was predicted to be deleterious (CADD Phred = 17.82).

**TABLE 4:** Genes with sex interactions (FDR *q* < 0.1) on neurodegenerative biomarkers in LASI-DAD.

Neurodegenerative biomarker	Gene	# of SNVs analyzed	cMAC	*P*-value	FDR *q*-value	ρ
Missense/loss-of-function SNVs in AD-associated genes						
Ab42	*EPHA1*	6	608	0.0022	0.094	0
tTau	*MS4A6A*	2	2,090	0.00053	0.024	1
Brain-specific promoter/enhancer SNVs in AD-associated genes						
tTau	*DGKQ*	12	8,636	0.0013	0.094	0.6
tTau	*MS4A6A*	7	2,570	0.0033	0.094	0
tTau	*MS4A4A*	4	781	0.0038	0.094	0
pTau	*JAZF1*	10	1,816	0.00047	0.034	0
Brain-specific promoter/enhancer SNVs in neurodegenerative biomarker-associated genes						
Ab40	*RNF214*	14	9,836	0.019	0.094	0.3

Ab42, amyloid β 42; AD, Alzheimer’s disease; cMAC, cumulative minor allele count; FDR, false discovery rate; pTau, tau phosphorylated at threonine 181; SNV, single nucleotide variant; tTau, total tau.

#### Brain-specific promoter/enhancer SNV analysis

A total of 76–77 AD-associated genes and all 25 neurodegenerative gene-biomarker pairs met the criteria for analysis. Three AD-associated genes (*DGKQ, MS4A6A, MS4A4A*) interacted with sex on tTau (*p* = 0.0013, *p* = 0.0033, *p* = 0.0038), and one AD-associated gene (*JAZF1*) interacted with sex on pTau (*p* = 0.00047) after correction for multiple testing (Table 4). One neurodegenerative biomarker gene (*RNF214*, *p* = 0.019, *q* = 0.094) also interacted with sex on Ab40 (Table 4).

The variants with the strongest interactions in the identified genes all tended to be rare or low-frequency, and most were associated with lower tTau or pTau in males only (Supplementary Table S8). Almost all were predicted to be deleterious (CADD Phred > 17). rs61742546, which had the smallest interaction *p*-value in both *MS4A4A* and *MS4A6A* in the promote/enhancer analysis, also appeared in missense/LoF analysis in *MS4A6A*. The SNV for *DGKQ* was associated with higher tTau in males only but lower tTau in females only (β_males_ = 0.13, β_females_ = −0.42). The sentinel SNV in *RNF214* had opposite effect directions across sexes and is also an intron variant in *PCSK7* (β_males_ = 0.19, β_females_ = −0.20) (Supplementary Table S9). The SNVs rs147073461 in *JAZF1*, rs571384095 in *DGKQ*, and rs369511965 in *RNF214* had higher MAFs in LASI-DAD compared to other global populations.

### Interaction between genes and age

#### Missense/LoF SNV analysis

A total of 72–73 AD-associated genes and 23 neurodegenerative gene-biomarker pairs met the criteria for analysis. One AD-associated gene (*APOE*) exhibited an interaction with age on Ab42 after correction for multiple testing (*p* = 0.00020, FDR *q* = 0.0040). Other genes that interacted with age included six genes on GFAP (*SORT1, TNIP1, BLNK, MADD, SORL1, MAPT*; *p* = 5.41 × 10^–5^ to 0.0039), five genes on NfL (*ADAM17, EPHA1-AS1, BLNK, RIN3, NTN5*; *p* = 0.00031–0.0072), one gene on tTau (*SPI1, p* = 8.55 × 10^–5^), and one gene on pTau (*NYAP1*, *p* = 0.00011) (Table 5). *APOE* was the only gene of the neurodegenerative gene-biomarker pairs that showed interaction with age on Ab42 after correction for multiple testing.

**TABLE 5:** Genes with significant age interactions (FDR *q* < 0.1) on neurodegenerative biomarkers in LASI-DAD.

Neurodegenerative biomarker	Gene	# of SNVs analyzed	cMAC	*P*-value	FDR *q*-value	ρ
Missense/loss-of-function SNVs in AD-associated genes						
Ab42	*APOE*	2	587	0.00020	0.011	0.6
tTau	*SPI1*	2	13	8.55E-05	0.0052	1
pTau	*NYAP1*	6	53	0.00011	0.0068	0.1
GFAP	*SORT1*	3	21	8.87E-05	0.0027	0.1
GFAP	*TNIP1*	6	500	0.00081	0.0098	0
GFAP	*BLNK*	3	25	5.41E-05	0.0027	0
GFAP	*MADD*	7	1,523	0.00027	0.0053	0.9
GFAP	*SORL1*	10	655	0.00074	0.0098	0
GFAP	*MAPT*	10	3,009	0.0039	0.039	1
NfL	*ADAM17*	2	61	0.0068	0.085	0
NfL	*EPHA1-AS1*	4	1,592	0.00031	0.018	1
NfL	*BLNK*	3	24	0.0040	0.079	0
NfL	*RIN3*	8	4,242	0.0072	0.085	1
NfL	*NTN5*	7	128	0.0037	0.079	0
Brain-specific promoter/enhancer SNVs in AD-associated genes						
GFAP	*TNIP1*	2	219	0.0027	0.099972	1
GFAP	*ZCWPW1*	22	4,352	8.17E-06	0.00061	0
Missense/loss-of-function SNVs in neurodegenerative biomarker-associated genes						
Ab42	*APOE*	2	587	0.00020	0.00039	0.6

Ab42, amyloid β 42; AD, Alzheimer’s disease; cMAC, cumulative minor allele count; FDR, false discovery rate; GFAP, glial fibrillary acidic protein; NfL, neurofilament light chain; pTau, tau phosphorylated at threonine 181; SNV, single nucleotide variant; tTau, total tau.

We extracted the SNVs with the strongest age interaction for all genes (Supplementary Tables S10, S11). Nearly all SNVs were predicted to be deleterious (CADD Phred > 10). Additionally, the main effects of nearly all identified variants were in the direction of higher neurodegenerative biomarkers (Ab42, tTau, pTau, GFAP, NfL), which are associated with higher AD risk in the case of tTau, pTau, GFAP, and NfL. For most age interactions, the genetic effects become more pronounced at higher ages (Supplementary Figure S9). The top variants in *NTN5, BLNK, RIN3, MADD*, and *NYAP1* had higher MAFs in LASI-DAD compared to other global populations.

**FIGURE 3:**
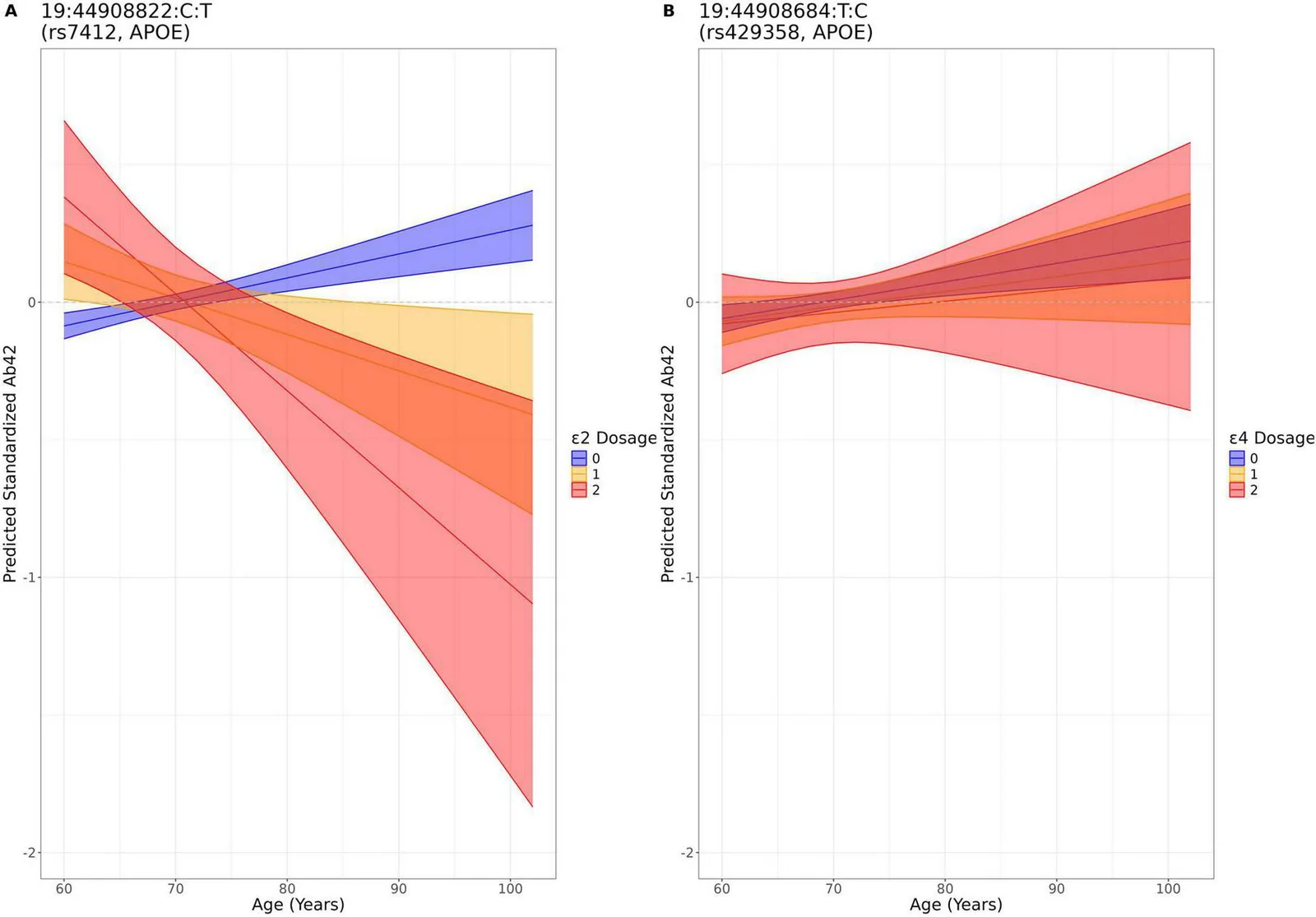
Plot of predicted standardized amyloid β 42 (Ab42) by age and **(A)** rs7412 minor allele T (ε2) and **(B)** rs429358 minor allele C (ε4) dosage across the age range of LASI-DAD. Predicted values were estimated from a model adjusting for age, sex, and the first 10 PCs of global ancestry. The dashed line shows the mean standardized Ab42 level. The bands represent 95% confidence intervals for the mean. Ab42 appears to increase with age in those without ε2, but decrease with age for those carrying at least one copy of ε2. ε4 does not modify the relationship between age and predicted standardized Ab42 levels.

Interestingly, an *APOE* interaction with age on Ab42 was significant, weighted more heavily toward the burden score (ρ = 0.6), even though the main effect between *APOE* and Ab42 was not. Since there are only two variants in the *APOE* gene available for missense/LoF analysis (ε2 and ε4), which are known to have opposite effects on AD risk (Farrer et al., 1997), we explored which variant was driving the interaction. To visualize how the effects of age on the biomarker differed by ε2 and ε4 carriership (determined by SNVs rs7412 and rs429358), we plotted predicted standardized Ab42 by age and rs7412 (ε2) or rs429358 (ε4) minor allele dosage (Figure 3). The ε2 allele was associated with higher Ab42 at younger ages, but lower Ab42 at older ages, while ε4 showed no interaction.

#### Brain-specific promoter/enhancer SNV analysis

A total of 76–77 AD-associated genes and all 25 neurodegenerative gene-biomarker pairs met the criteria for analysis. Two AD-associated genes (*ZCWPW1, TNIP1*) had interactions with age on GFAP after correction for multiple testing (*p* = 0.0027, *p* = 8.17 × 10^–6^, respectively) (Table 5). No neurodegenerative biomarker genes had interactions with age after correction for multiple testing. The variant with the smallest interaction *p*-value in *ZCWPW1*, rs537662968, does not appear in any other global population, and is also an intron variant in *MEPCE* (Supplementary Figure S10 and Supplementary Tables S10). As above, for both age interactions, the genetic effects become more pronounced at higher ages (Supplementary Figure S10).

## Discussion

Plasma neurodegenerative biomarkers of Alzheimer’s disease have been associated with cognitive performance in multiple populations, including South Asians (Kim et al., 2025; Márquez et al., 2025; Jiang et al., 2026). However, genetic associations with these biomarkers are largely unknown, particularly in non-Europeans. We performed a gene-based analysis using 84 genes identified from AD GWASs and 25 gene-biomarker pairs identified from previous neurodegenerative biomarker GWASs for associations with seven neurodegenerative biomarkers in South Asian participants living in India. We found that missense/LoF variants in six AD-associated genes (*CLU, APOE, ICA1L, CASS4, EPHA1, ECHDC3*) and brain-specific promoter and enhancer variants in one gene (*CCD6*) were associated with at least one neurodegenerative biomarker in LASI-DAD. No previously identified neurodegenerative biomarker genes were associated with biomarkers in LASI-DAD beyond *APOE* and *APOC1*. We also found that missense/LoF variants in *EPHA1*, which were associated with GFAP in our primary analysis, had an interaction with sex on Ab42. Sex interactions were also present for promoter/enhancer SNVs on tTau (*DGKQ, MS4A6A*, and *MS4A4A*) and Ab40 (*RNF214*). A total of 14 AD-associated genes interacted with age for at least one neurodegenerative biomarker, including *APOE*, the strongest known genetic risk factor for AD, and *MAPT*, the gene that encodes the tau protein.

Nearly all variants with the strongest interactions within each identified gene were predicted to be deleterious. Some were associated with higher neurodegenerative biomarkers at younger ages that attenuated with higher age, and others showed the opposite pattern. In either case, the genetic effects were more pronounced at older ages. Several of the top SNVs in the gene-based analyses and the interaction analyses had higher frequencies in the LASI-DAD analytic sample compared to NFE, EAS, AFR, and MID populations. This study highlights variants associated with AD risk and progression within the Indian population, which has been historically understudied. These results may be important for precision health in South Asians across India and expatriates from India, as variants higher in frequency in South Asia compared to global populations, including those potentially modified by demographic variables, may play a role in shaping neurodegeneration as measured by the biomarkers. AD risk stratification in South Asians across India may need to account for such variant effects.

We found that *APOE* and *CLU* missense SNVs were associated with Ab42/Ab40. This is not unexpected, since *APOE* directly influences β-amyloid metabolism (Kanekiyo et al., 2014; Xia et al., 2024). Given that the *APOE* ε2 and ε4 alleles were associated with lower (Conejero-Goldberg et al., 2014) and higher (Corder et al., 1993) AD risk relative to ε3, respectively, it is unsurprising to find that rs429358 (ε4) was associated with lower blood Ab42/Ab40, which has been implicated in dementia progression (Pérez-Grijalba et al., 2019). *CLU* produces clusterin protein, which complexes with Ab42 to prevent aggregation into β-amyloid plaques (Kim et al., 2022). Both are also regulated by some of the same genes, including *SORL1* (Lee A. et al., 2023). *ICAIL* and *CASS4* showed associations with pTau in our study. *ICA1L* has been associated with small vessel strokes (Cullell et al., 2022), as well as LATE-NC (Yu et al., 2022), an AD pathology that increases cognitive decline and is related to tau pathologies (Tomé et al., 2023). *CASS4* was previously associated with tau metabolism (Karch and Goate, 2014). We also found an association between *EPHA1* missense variants and GFAP, a generalized marker of neuroinflammation that encodes signaling receptors on T lymphocytes (Aasheim et al., 2005). Although these associations are consistent with biological expectations, the effects of individual SNVs across the identified genes were not always in the expected direction. For example, some of the identified missense/LoF variants predicted to be deleterious may have protective effects on AD (e.g., lower levels of pTau and tTau). One explanation for this may be that the variants result in an mRNA or protein product that is quickly degraded by the cell, leading to decreased biomarker levels. Alternatively, these variants may disrupt protein function in a way that lowers protein concentration but is overall deleterious for the individual.

Overall, we found that missense/LoF variants in AD-associated genes were more likely to be associated with neurodegenerative biomarkers than promoter/enhancer variants. This may be due to the larger impact of protein altering mutations on biomarker levels compared to the subtler effects of regulatory SNVs. The missense/LoF SNVs also tend to have larger effect estimates and higher CADD Phred scores compared to the promoter/enhancer SNVs.

We identified several AD-associated genes that showed interactions with sex on tTau, pTau, and Ab42. In several genes, the minor alleles of the sentinel SNVs were associated with lower Ab42, pTau, and tTau in males. For example, the minor allele of rs6967117 (T) in *EPHA1* was associated with lower Ab42 in males and higher Ab42 in females. It is known that females tend to have higher levels of tTau potentially due to differences in sex hormone levels (Sundermann et al., 2020). *MS4A6A*, one of the genes that showed sex differences, is part of the *MS4A* gene cluster which signals immune cell activation (Proitsi et al., 2014) which is also modulated by sex hormones (Taneja, 2018). Additionally, lower tTau levels in LASI-DAD males at older ages compared to expectation could reflect selective survival.

We also found that several genes, including *APOE*, had an interaction with age, primarily on GFAP and NfL, generalized markers of inflammation that increase with age (Borko et al., 2023). Interaction effects were mixed, with some minor alleles associated with deleterious effects (higher biomarker levels) and others with protective effects (lower biomarker levels). In most cases, the genetic effects increased with age. *APOE* showed an interaction on Ab42 which was driven by the ε2 variant in which ε2 was associated with higher Ab42 at younger ages, but lower Ab42 at older ages. A previous study also showed that ε2-carriership is associated with reduced age-related accumulation of Ab42 in the brain (Insel et al., 2021). In contrast, the lack of an ε4 carriership by age interaction is in contrast to a previous study which found that ε4-carriership blunts the association between higher age and increased blood Ab42 (Nakamura et al., 2018); however, the age of participants in this study (over 18 years) was much more variable than in our sample. Further, the effect of ε4 may peak around 80 years old, after which the effects of other genes may become stronger (Bellou et al., 2020). As the median age in our cohort is 68, we may not have been able to detect this interaction, as most of our participants are younger than 80 years.

Our study had several strengths. Gene-based analysis allowed us to aggregate SNVs within genes and detect associations and interactions with rare or uncommon variants. Our analysis of missense/LoF SNVs and brain-specific promoter/enhancer SNVs placed focus on identifying associations with functional variants. Our study of neurodegenerative biomarkers, rather than downstream phenotypes such as cognitive function or AD, helped identify variants more closely linked with AD development and pathology. Our study also focused on South Asians from India, which has a unique genetic composition compared to other populations. Rare and low-frequency variants may have risen to higher frequencies in India due to founder events and endogamous mating. Further, the South Indian component of genetic ancestry is unique to India (Kerdoncuff et al., 2025).

Our study haslimitations. First, we could not study insertion/deletions (indels) due to the lack of available annotation scores. Indels may have a stronger impact on gene products compared to SNVs, and their removal may have attenuated gene associations and interactions on neurodegenerative biomarkers. We also focused on only two functional classes of SNVs, yet other classes such as splice site variants may have important effects on these biomarkers. Another limitation is that to improve statistical properties, we Winsorized the biomarker measurements, which may have resulted in the dampening of signal from variants with more extreme effects. We were also not able to analyze any previously associated biomarker genes for GFAP and NfL due to the lack of large GWAS for these biomarkers. The GWASs that did inform this study were conducted primarily or exclusively among EA participants. However, it is possible that different LD structure and allele frequencies between populations, as well as different environmental factors, may have attenuated the ability to detect associations. Further, given that power to detect rare variant associations is limited with moderate sample sizes such as ours, the findings presented here must be replicated in larger sample sizes and in other South Asian populations to be considered robust and representative. We were also limited to the examination of previously identified genes, so future studies will be needed to identify novel genetic associations across the genome in South Asians. Population genetic studies may help to discern whether the identified variants likely originated in South India. Finally, sample size prevented us from directly testing whether the identified variants influenced dementia risk within our study.

In conclusion, several genes previously associated with amyloid β metabolism, tau metabolism, and immune response were associated with neurodegenerative biomarkers in this South Asian sample. We also identified genes that had different effects depending on demographic variables. The minor alleles of variants in genes with sex-specific effects tended to be associated primarily with biomarker changes that correlate with lower AD risk in males compared to females. We also identified genes that had stronger effects on neurodegenerative biomarkers at older ages. The identified genes harbored some variants with potentially deleterious effects, several of which may be enriched in India compared to other global populations.

## Data Availability

Harmonized Diagnostic Assessment of Dementia for the Longitudinal Aging Study in India (LASI-DAD) whole genome sequencing data may be accessed at the National Institute on Aging Genetics of Alzheimer’s Disease Data Storage Site (NIAGADS), with accession number NG00067–ADSP Umbrella (https://dss.niagads.org/datasets/ng00067/). Phenotype data may be accessed at the Gateway to Global Aging website, https://g2aging.org/.
