# Supplementary material for: Association between Alzheimer’s disease-related genes and neurodegenerative blood-based biomarkers in Indian adults
Source: Front Aging Neurosci. 2026 Aug 19;18:1832155. doi: 10.3389/fnagi.2026.1832155 (PMC13534049; doi:10.3389/fnagi.2026.1832155)
Supplement: Supplementary file 1 [file Supplementary_file_1.docx]

**Supplemental Materials**

***Supplemental Tables***

**Table S1:** AD-associated genes identified from GWAS

| Gene | Locus | Source |
| --- | --- | --- |
| *ABCA1* | *ABCA1* | Bellenguez et al. (2022) |
| *ABCA7* | *ABCA7* | Wightman et al. (2021) and Bellenguez et al. (2022) |
| *ABI3* | *ABI3* | Wightman et al. (2021) and Bellenguez et al. (2022) |
| *ACE* | *ACE* | Wightman et al. (2021) and Bellenguez et al. (2022) |
| *ADAM10* | *ADAM10* | Wightman et al. (2021) and Bellenguez et al. (2022) |
| *ADAM17* | *ADAM17* | Bellenguez et al. (2022) |
| *ADAMTS1* | *ADAMTS1* | Bellenguez et al. (2022) |
| *AGRN* | *AGRN* | Wightman et al. (2021) |
| *APH1B* | *APH1B* | Wightman et al. (2021) and Bellenguez et al. (2022) |
| *APOC1* | *APOE* | None |
| *APOE* | *APOE* | Wightman et al. (2021) |
| *APP* | *APP* | Wightman et al. (2021) and Bellenguez et al. (2022) |
| *BIN1* | *BIN1* | Wightman et al. (2021) and Bellenguez et al. (2022) |
| *BLNK* | *BLNK* | Bellenguez et al. (2022) |
| *CASS4* | *CASS4* | Wightman et al. (2021) and Bellenguez et al. (2022) |
| *CCDC6* | *ANK3* | Wightman et al. (2021) and Bellenguez et al. (2022) |
| *CD2AP* | *CD2AP* | Wightman et al. (2021) and Bellenguez et al. (2022) |
| *CD33* | *CD33* | Wightman et al. (2021) |
| *CELF1* | *CELF1/SPI1* | Bellenguez et al. (2022) |
| *CLNK* | *CLNK/HS3ST1* | Wightman et al. (2021) and Bellenguez et al. (2022) |
| *CLU* | *CLU* | Wightman et al. (2021) and Bellenguez et al. (2022) |
| *CR1* | *CR1* | Wightman et al. (2021) and Bellenguez et al. (2022) |
| *CTSB* | *CTSB* | Bellenguez et al. (2022) |
| *CTSH* | *CTSH* | Bellenguez et al. (2022) |
| *DGKQ* | *IDUA* | Bellenguez et al. (2022) |
| *DOC2A* | *DOC2A* | Bellenguez et al. (2022) |
| *ECHDC3* | *USP6NL/ECHDC3* | Wightman et al. (2021) and Bellenguez et al. (2022) |
| *EGFR* | *SEC61G* | Bellenguez et al. (2022) |
| *EPHA1* | *EPHA1/EPHA1-AS1* | Bellenguez et al. (2022) |
| *EPHA1-AS1* | *EPHA1/EPHA1-AS1* | Wightman et al. (2021) |
| *FERMT2* | *FERMT2* | Wightman et al. (2021) and Bellenguez et al. (2022) |
| *GRN* | *GRN* | Wightman et al. (2021) and Bellenguez et al. (2022) |
| *HAVCR2* | *HAVCR2* | Wightman et al. (2021) |
| *HLA-DQA1* | *HLA* | Wightman et al. (2021) and Bellenguez et al. (2022) |
| *HLA-DRB1* | *HLA* | Wightman et al. (2021) and Bellenguez et al. (2022) |
| *HLA-DRB5* | *HLA* | Wightman et al. (2021) and Bellenguez et al. (2022) |
| *HS3ST1* | *CLNK/HS3ST1* | Bellenguez et al. (2022) |
| *ICA1* | *ICA1* | Bellenguez et al. (2022) |
| *ICA1L* | *WDR12* | Bellenguez et al. (2022) |
| *IL34* | *IL34* | Bellenguez et al. (2022) |
| *INPP5D* | *INPP5D* | Wightman et al. (2021) and Bellenguez et al. (2022) |
| *JAZF1* | *JAZF1* | Bellenguez et al. (2022) |
| *KAT8* | *KAT8* | Bellenguez et al. (2022) |
| *LILRB2* | *LILRB2* | Wightman et al. (2021) and Bellenguez et al. (2022) |
| *LIME1* | *SLC2A4RG* | Bellenguez et al. (2022) |
| *MADD* | *CELF1/SPI1* | Wightman et al. (2021) and Bellenguez et al. (2022) |
| *MAF* | *MAF* | Bellenguez et al. (2022) |
| *MAPT* | *MAPT* | Bellenguez et al. (2022) |
| *MME* | *MME* | Bellenguez et al. (2022) |
| *MS4A4A* | *MS4A* | Wightman et al. (2021) and Bellenguez et al. (2022) |
| *MS4A6A* | *MS4A* | Wightman et al. (2021) and Bellenguez et al. (2022) |
| *MYO15A* | *MYO15A* | Bellenguez et al. (2022) |
| *NCK2* | *NCK2* | Wightman et al. (2021) and Bellenguez et al. (2022) |
| *NME8* | *NME8* | Bellenguez et al. (2022) |
| *NTN5* | *NTN5* | Wightman et al. (2021) |
| *NYAP1* | *ZCWPW1/NYAP1* | Wightman et al. (2021) and Bellenguez et al. (2022) |
| *OTULIN* | *ANKH* | Bellenguez et al. (2022) |
| *PICALM* | *PICALM* | Wightman et al. (2021) and Bellenguez et al. (2022) |
| *PLCG2* | *PLCG2* | Bellenguez et al. (2022) |
| *PLEKHA1* | *PLEKHA1* | Bellenguez et al. (2022) |
| *PTK2B* | *PTK2B* | Bellenguez et al. (2022) |
| *RABEP1* | *SCIMP/RABEP1* | Wightman et al. (2021) and Bellenguez et al. (2022) |
| *RBCK1* | *RBCK1* | Bellenguez et al. (2022) |
| *RHOH* | *RHOH* | Bellenguez et al. (2022) |
| *RIN3* | *SLC24A4/RIN3* | Wightman et al. (2021) and Bellenguez et al. (2022) |
| *RITA1* | *TPCN1* | Bellenguez et al. (2022) |
| *SCIMP* | *SCIMP/RABEP1* | Wightman et al. (2021) and Bellenguez et al. (2022) |
| *SHARPIN* | *SHARPIN* | Wightman et al. (2021) and Bellenguez et al. (2022) |
| *SIGLEC11* | *SIGLEC11* | Bellenguez et al. (2022) |
| *SLC24A4* | *SLC24A4/RIN3* | Bellenguez et al. (2022) |
| *SORL1* | *SORL1* | Wightman et al. (2021) and Bellenguez et al. (2022) |
| *SORT1* | *SORT1* | Bellenguez et al. (2022) |
| *SPI1* | *CELF1/SPI1* | Wightman et al. (2021) and Bellenguez et al. (2022) |
| *SPPL2A* | *SPPL2A* | Bellenguez et al. (2022) |
| *TMEM106B* | *TMEM106B* | Wightman et al. (2021) and Bellenguez et al. (2022) |
| *TNIP1* | *TNIP1* | Wightman et al. (2021) and Bellenguez et al. (2022) |
| *TOMM40* | *APOE* | None |
| *TREM2* | *TREM2* | Wightman et al. (2021) and Bellenguez et al. (2022) |
| *TSPAN14* | *TSPAN14* | Bellenguez et al. (2022) |
| *TSPOAP1* | *TSPOAP1/TSPOAP1-AS1* | Bellenguez et al. (2022) |
| *TSPOAP1-AS1* | *TSPOAP1/TSPOAP1-AS1* | Wightman et al. (2021) |
| *USP6NL* | *USP6NL/ECHDC3* | Wightman et al. (2021) |
| *WDR81* | *WDR81* | Bellenguez et al. (2022) |
| *ZCWPW1* | *ZCWPW1/NYAP1* | Wightman et al. (2021) and Bellenguez et al. (2022) |

Adapted from Abu-Amara et al. (2025) Supplemental Table 1.

**Table S2:** Neurodegenerative biomarker GWAS used for gene selection

| Source | Sample size | Ancestry | Biomarkers | # Genes selected | Selection criteria |
| --- | --- | --- | --- | --- | --- |
| Jansen et al. (2022) | N=8074 in discovery stage, N=5042 in replication stage | European | Cerebrospinal fluid Ab42, pTau, tTau | 6 | Selected genes nearest to the variants with p<5x10^-8^ in meta-analysis. |
| Damotte et al.(2021) | N=12,396 | European | Plasma Ab40, Ab42, Ab42/Ab40 | 10 | Selected genes with p<2.76x10^-6^ in gene-based analysis of single-nucleotide variants (SNVs) within defined gene boundaries using MAGMA (See Table 2 of Damotte et al. (2021)). |
| Sarnowksi et al. (2022) | N=14,721 European ancestry,  N=953 African ancestry | European, African | Plasma/serum tTau | 10 | Selected genes with p≤1.25x10^-6^ in gene-based analysis (SKAT) with variants annotated from whole-exome data. |

Ab40=amyloid β 40, Ab42=amyloid β 42, Ab42/Ab40= Ab42 to Ab40 ratio, pTau=tau phosphorylated at threonine 181, tTau=total tau

**Table S3:** Pairs of genes and neurodegenerative biomarkers previously associated in GWAS

| Gene | Biomarker | Study |
| --- | --- | --- |
| *APOE* | Ab42 | Jansen et al. (2022) |
| *CR1* | A42 | Jansen et al. (2022) |
| *APOE* | pTau | Jansen et al. (2022) |
| *BIN1* | pTau | Jansen et al. (2022) |
| *GMNC* | pTau | Jansen et al. (2022) |
| *C16orf95* | pTau | Jansen et al. (2022) |
| *PSEN2* | Ab40 | Damotte et al.(2021) |
| *CCK* | Ab40 | Damotte et al.(2021) |
| *RNF214* | Ab40 | Damotte et al.(2021) |
| *BACE1* | Ab40 | Damotte et al.(2021) |
| *APP* | Ab40 | Damotte et al.(2021) |
| *APOE* | Ab42 | Damotte et al.(2021) |
| *APOC1* | Ab42 | Damotte et al.(2021) |
| *ZNF397* | Ab42/Ab40 | Damotte et al.(2021) |
| *APOE* | Ab42/Ab40 | Damotte et al.(2021) |
| *APOC1* | Ab42/Ab40 | Damotte et al.(2021) |
| *ELFN2* | tTau | Sarnowksi et al. (2022) |
| *UBASH3B* | tTau | Sarnowksi et al. (2022) |
| *RUSF1* | tTau | Sarnowksi et al. (2022) |
| *ZFP28* | tTau | Sarnowksi et al. (2022) |
| *LCT* | tTau | Sarnowksi et al. (2022) |
| *REM1* | tTau | Sarnowksi et al. (2022) |
| *DELE1* | tTau | Sarnowksi et al. (2022) |
| *SLIT3* | tTau | Sarnowksi et al. (2022) |
| *NSD3* | tTau | Sarnowksi et al. (2022) |
| *MYO1G* | tTau | Sarnowksi et al. (2022) |

Ab40=amyloid β 40, Ab42= amyloid β 42, Ab42/Ab40=Ab42 to Ab40 ratio, pTau= tau phosphorylated at threonine 181, tTau=total tau.

**Table S4:** Summary of variant counts and functional annotations across gene sets for participants that had measures of tTau (N=2,192)

| SNV Type | Minimum | Q1 | Median | Q3 | Maximum | Mean | SD |
| --- | --- | --- | --- | --- | --- | --- | --- |
| *AD-associated genes* | | | | | | | |
| Missense/LoF | 2 | 3 | 6 | 8 | 39 | 7.21 | 6.67 |
| Missense | 2 | 3 | 6 | 8 | 37 | 7.08 | 6.49 |
| LoF | 1 | 1 | 1 | 1.75 | 2 | 1.33 | 0.516 |
| Promoter/Enhancer | 2 | 14 | 22 | 32 | 76 | 24.8 | 51.97 |
| Promoter | 2 | 14 | 21.5 | 29.8 | 71 | 23.1 | 13.4 |
| Enhancer | 5 | 8.5 | 12 | 14.5 | 76 | 17.6 | 20.1 |
| *Neurodegenerative biomarker-associated genes* | | | | | | | |
| Missense/LoF | 2 | 2 | 3 | 7 | 17 | 5.47 | 4.88 |
| Missense | 2 | 2 | 3 | 7 | 17 | 5.41 | 4.76 |
| LoF | 1 | 1 | 1 | 1 | 1 | 1 | N/A |
| Promoter/Enhancer | 4 | 13.2 | 18.5 | 22.5 | 65 | 19.8 | 13.1 |
| Promoter | 4 | 14 | 18 | 21 | 65 | 19.5 | 12.8 |
| Enhancer | 9 | 10.8 | 12.5 | 14.2 | 16 | 12.5 | 4.95 |

AD=Alzheimer’s disease, LoF=loss-of-function, Q1=quartile 1, Q3=quartile 3, SD=standard deviation, SNV=single nucleotide variant, tTau=total tau

**Table S5:** Annotation score weight summaries of missense/LoF SNVs in gene-based analysis for tTau (N=2,192)

| Annotation | Minimum | Q1 | Median | Q3 | Maximum | Mean | SD |
| --- | --- | --- | --- | --- | --- | --- | --- |
| *AD-associated genes* | | | | | | | |
| CADD_raw_rankscore | 0.00 | 0.71 | 0.98 | 1.00 | 1.00 | 0.77 | 0.36 |
| Fathmm-MKL-coding_rankscore | 1.93x10^-3^ | 0.66 | 0.95 | 0.99 | 1.00 | 0.78 | 0.30 |
| GERP_RS_rankscore | 5.00x10^-5^ | 0.36 | 0.91 | 0.98 | 1.00 | 0.70 | 0.37 |
| Eigen.Phred (Transformed) | 1.74x10^-5^ | 0.07 | 0.24 | 0.49 | 0.98 | 0.31 | 0.27 |
| *Neurodegenerative biomarker-associated genes* | | | | | | | |
| CADD_raw_rankscore | 0.00 | 0.78 | 0.98 | 1.00 | 1.00 | 0.83 | 0.28 |
| Fathmm-MKL-coding_rankscore | 3.90x10^-3^ | 0.77 | 0.95 | 0.99 | 1.00 | 0.82 | 0.26 |
| GERP_RS_rankscore | 1.50x10^-3^ | 0.40 | 0.95 | 0.99 | 1.00 | 0.73 | 0.36 |
| Eigen.Phred (Transformed) | 1.74x10^-5^ | 0.08 | 0.25 | 0.46 | 0.96 | 0.30 | 0.25 |

AD=Alzheimer’s disease, LoF=loss-of-function, Q1=quartile 1, Q3=quartile 3, SD=standard deviation, SNV=single nucleotide variant, tTau=total tau

**Table S6:** Annotation score weight summaries of brain-specific promoter/enhancer SNVs in gene-based analysis for tTau (N=2,192)

| Annotation | Minimum | Q1 | Median | Q3 | Maximum | Mean | SD |
| --- | --- | --- | --- | --- | --- | --- | --- |
| AD-associated genes | | | | | | | |
| CADD_raw_rankscore | 0.00 | 0.38 | 0.73 | 0.88 | 1.00 | 0.63 | 0.31 |
| Fathmm-MKL-noncoding_rankscore | 0.00 | 0.51 | 0.75 | 0.89 | 1.00 | 0.67 | 0.26 |
| GERP_RS_rankscore | 0.00 | 0.12 | 0.40 | 0.82 | 1.00 | 0.47 | 0.35 |
| Eigen.PC.Phred (Transformed) | 0.00 | 0.91 | 0.98 | 0.99 | 1.00 | 0.89 | 0.21 |
| GenoCanyon_rankscore | 0.60 | 0.93 | 0.97 | 0.97 | 1.00 | 0.94 | 0.05 |
| Neurodegenerative biomarker-associated genes | | | | | | | |
| CADD_raw_rankscore | 0.00 | 0.36 | 0.69 | 0.86 | 1.00 | 0.61 | 0.30 |
| Fathmm-MKL-noncoding_rankscore | 0.00 | 0.46 | 0.72 | 0.87 | 0.99 | 0.64 | 0.27 |
| GERP_RS_rankscore | 0.00 | 0.12 | 0.39 | 0.81 | 0.99 | 0.46 | 0.34 |
| Eigen.Phred (Transformed) | 0.01 | 0.80 | 0.95 | 0.99 | 1.00 | 0.84 | 0.24 |
| GenoCanyon_rankscore | 0.12 | 0.90 | 0.97 | 0.97 | 1.00 | 0.91 | 0.12 |

AD=Alzheimer’s disease, Q1=quartile 1, Q3=quartile 3, SD=standard deviation, SNV=single nucleotide variant, tTau=total tau

**Table S7:** Sensitivity analysis of significantly-associated (FDR q<0.1) AD- and neurodegenerative-biomarker associated genes after additional adjustment for state/territory

| Variant Set | Gene Set | Biomarker | Gene | P-value (With Annotations) - Original | P-value (With Annotations) - State Adjusted | P-value (Without Annotations) - Original | P-value (Without Annotations) - State Adjusted |
| --- | --- | --- | --- | --- | --- | --- | --- |
| Missense/LoF | AD Associated | Ab40 | *ECHDC3* | 0.001229 | 0.0004978 | - | - |
| Missense/LoF | AD Associated | Ab42/Ab40 | *CLU* | - | - | 0.003369 | 0.004835 |
| Missense/LoF | AD Associated | Ab42/Ab40 | *APOE* | - | - | 0.002004 | 0.001368 |
| Missense/LoF | AD Associated | GFAP | *EPHA1* | 9.608x10^-6^ | 1.867x10^-5^ | 3.757x10^-5^ | 5.621x10^-5^ |
| Missense/LoF | AD Associated | pTau | *ICA1L* | - | - | 0.002959 | 0.002907 |
| Missense/LoF | AD Associated | pTau | *CASS4* | - | - | 0.00298 | 0.004865 |
| Promoter/Enhancer | AD Associated | Ab42/Ab40 | *CCDC6* | 0.001114 | 0.003224 | 0.001206 | 0.003278 |
| Missense/LoF | Neurodegenerative Biomarker Associated | Ab42/Ab40 | *APOE* | 0.001977 | 0.001336 | 0.002004 | 0.001368 |
| Promoter/Enhancer | Neurodegenerative Biomarker Associated | Ab42/Ab40 | *APOE* | - | - | 0.05461 | 0.05330 |
| Promoter/Enhancer | Neurodegenerative Biomarker Associated | Ab42/Ab40 | *APOC1* | - | - | 0.04686 | 0.02978 |

P-value in original model was calculated from STAAR tests with a null model adjusting for age, sex, the first 10 Principal Components of global ancestry, and a random intercept for genetic relatedness. P-value in state-adjusted model additionally adjusted for state/territory. Gene p-values were only calculated for tests where the FDR q<0.1. A dash indicates FDR q≥0.1 in that specific test.

Ab40=amyloid β 40, Ab42=amyloid β 42, Ab42/Ab40=Ab42 to Ab40 ratio, AD=Alzheimer’s Disease, FDR=false discovery rate, GFAP=glial fibrillary acidic protein, LoF=loss-of-function, pTau=tau phosphorylated at threonine 181.

**Table S8:** Sex-specific effect estimates for AD-associated genes that had interactions with sex

| Analysis | Neurodegenerative  Biomarker | ID | Gene | GxE p | Minor Allele | MAC | MAF  (Analytic) | CADD  Phred | AF  (EAS) | AF  (AFR) | AF  (MID) | AF  (NFE) | AF  (SAS) | Male Effect (95% CI) | Female Effect (95%CI) |
| --- | --- | --- | --- | --- | --- | --- | --- | --- | --- | --- | --- | --- | --- | --- | --- |
| Missense/LoF | Ab42 | 7:143391774:T:C (rs6967117) | *EPHA1* | 0.00078 | T | 61 | 0.017 | 11.4 | 0.0014 | 0.085 | 0.014 | 0.077 | 0.014 | **‑0.30  (‑0.52, ‑0.07)** | **0.22**  **(0.02, 0.42)** |
| Missense/LoF | tTau | 11:60181585:A:G  (rs61742546) | *MS4A6A* | 0.00051 | G | 27 | 0.0062 | 17.82 | 0 | 0.0045 | 0.027 | 0.024 | 0.0091 | **‑0.54  (‑0.90, ‑0.17)** | 0.26  (**‑**0.00, 0.51) |
| Promoter/Enhancer | tTau | 4:984935:C:T  (rs571384095) | *DGKQ* | 0.0094 | T | 32 | 0.0073 | 3.63 | 0 | 2.41E-05 | 0 | 8.82E-05 | 0.0043 | 0.13  (-0.11, 0.37) | **-0.42**  **(-0.75, -0.08)** |
| Promoter/Enhancer | tTau | 11:60181585:A:G (rs61742546) | *MS4A6A* | 0.00051 | G | 27 | 0.0062 | 17.82 | 0 | 0.0045 | 0.027 | 0.024 | 0.0091 | **‑0.54  (‑0.90, ‑0.17)** | 0.26  (**‑**0.00, 0.51) |
| Promoter/Enhancer | tTau | 11:60181585:A:G (rs61742546) | *MS4A4A* | 0.00051 | G | 27 | 0.0062 | 17.82 | 0 | 0.0045 | 0.027 | 0.024 | 0.0091 | **‑0.54  (‑0.90, ‑0.17)** | 0.26  (**‑**0.00, 0.51) |
| Promoter/Enhancer | pTau | 7:28180952:T:C (rs147073461) | *JAZF1* | 0.00084 | C | 67 | 0.015 | 17.73 | 0.00019 | 0.00079 | 0.0034 | 0.0047 | 0.011 | **‑0.34  (‑0.55, ‑0.12)** | 0.15  (**‑**0.04, 0.34) |

Ab42=amyloid β 42, AD=Alzheimer’s disease, AF=allele frequency, AFR=African, CI=confidence interval, EAS=East Asian, MAC=minor allele count, MAF=minor allele frequency, MID=Middle Eastern, NFE=non-Finnish European, pTau=tau phosphorylated at threonine 181, SAS=South Asian, tTau=total tau

**Table S9:** Sex-specific effect estimates for neurodegenerative biomarker-associated genes that had interactions with sex

| Analysis | Neurodegenerative  Biomarker | ID | Gene | GxE p | Minor Allele | MAC | MAF  (Analytic) | CADD  Phred | AF  (EAS) | AF  (AFR) | AF  (MID) | AF  (NFE) | AF  (SAS) | Male Effect (95% CI) | Female Effect (95%CI) |
| --- | --- | --- | --- | --- | --- | --- | --- | --- | --- | --- | --- | --- | --- | --- | --- |
| Promoter/Enhancer | Ab40 | 11:117230756:G:A (rs369511965) | *RNF214* | 0.00084 | A | 35 | 0.0083 | 1.50 | 0 | 0 | 0 | 4.41E-05 | 0.0050 | 0.19  (-0.06, 0.44) | -0.20  (**‑**0.62, 0.01) |

Ab40=amyloid β 40, AD=Alzheimer’s disease, AF=allele frequency, AFR=African, CI=confidence interval, EAS=East Asian, MAC=minor allele count, MAF=minor allele frequency, MID=Middle Eastern, NFE=non-Finnish European, pTau=tau phosphorylated at threonine 181, SAS=South Asian, tTau=total tau

**Table S10:** Estimates for the age effect and gene-by-age interaction effect for AD genes

| Analysis | Neurodegenerative Biomarker | ID | Gene | GxE p | Minor Allele | MAC | MAF  (Analytic) | AF  (EAS) | AF  (AFR) | AF  (MID) | AF  (NFE) | AF  (SAS) | CADD  Phred | Main  Effect | Interaction  Effect |
| --- | --- | --- | --- | --- | --- | --- | --- | --- | --- | --- | --- | --- | --- | --- | --- |
| Missense/LoF | Ab42 | 19:44908822:C:T  (rs7412) | *APOE* | 0.00022 | T | 182 | 0.050 | 0.080 | 0.10 | 0.048 | 0.079 | 0.037 | 26 | 1.55 | -0.02 |
| Missense/LoF | tTau | 11:47358871:C:T (rs75402116) | *SPI1* | 0.0033 | T | 8 | 0.0018 | 0.032 | 0.00026 | 0 | 0.00038 | 0.0023 | 18.06 | 4.28 | -0.06 |
| Missense/LoF | pTau | 7:100488878:C:T (rs376546614) | *NYAP1* | 9.06x10^-6^ | T | 15 | 0.0034 | 0 | 0.0015 | 0 | 1.47E-05 | 0.0023 | 12.92 | 6.27 | **‑**0.09 |
| Missense/LoF | GFAP | 1:109336281:C:G (rs2228606) | *SORT1* | 3.21x10^-5^ | G | 8 | 0.0018 | 0 | 0.0025 | 0.037 | 0.016 | 0.0033 | 21.5 | 13.39 | **‑**0.20 |
| Missense/LoF | GFAP | 5:151056942:G:C (rs2233290) | *TNIP1* | 6.09x10^-5^ | C | 187 | 0.043 | 0.00077 | 0.19 | 0.12 | 0.089 | 0.039 | 21.6 | 1.97 | **‑**0.03 |
| Missense/LoF | GFAP | 10:96223879:C:A (rs148612299) | *BLNK* | 0.00017 | A | 9 | 0.0021 | 0 | 0.0010 | 0.044 | 0.0049 | 0.0010 | 0.07 | 7.45 | **‑**0.11 |
| Missense/LoF | GFAP | 11:47285007:G:T  (rs557907564) | *MADD* | 1.17x10^-5^ | T | 5 | 0.0011 | 0 | 0 | 0 | 0 | 0.00083 | 3.14 | -14.03 | 0.18 |
| Missense/LoF | GFAP | 11:121478124:G:A (rs528123842) | *SORL1* | 1.33x10^-5^ | A | 8 | 0.0018 | 0.00039 | 0 | 0 | 1.47E-05 | 0.0015 | 24.7 | 9.62 | **‑**0.15 |
| Missense/LoF | GFAP | 17:45983657:G:A  (rs62063786) | *MAPT* | 0.0094 | A | 224 | 0.052 | 0.0016 | 0.049 | 0.21 | 0.22 | 0.074 | 6.57 | -1.17 | 0.02 |
| Missense/LoF | NfL | 2:9492963:C:T (rs61754177) | *ADAM17* | 0.0038 | T | 12 | 0.0028 | 0.00058 | 0.0029 | 0 | 0.0088 | 0.0021 | 25.7 | **‑**5.31 | 0.09 |
| Missense/LoF | NfL | 7:143408735:C:T  (rs79587607) | *EPHA1-AS1* | 0.0011 | T | 196 | 0.047 | 0.26 | 0.0038 | 0.022 | 0.0093 | 0.047 | 19.44 | 1.31 | -0.02 |
| Missense/LoF | NfL | 10:96204068:A:G (rs144266674) | *BLNK* | 0.00085 | G | 7 | 0.0016 | 0.00019 | 0.00017 | 0 | 0.00076 | 0.0012 | 20.5 | **‑**8.96 | 0.12 |
| Missense/LoF | NfL | 14:92577441:G:A  (rs2274542) | *RIN3* | 0.023 | A | 172 | 0.041 | 0.075 | 0.00022 | 0 | 0.00050 | 0.030 | 8.77 | 1.16 | -0.02 |
| Missense/LoF | NfL | 19:48661721:G:C (rs533570940) | *NTN5* | 6.09x10^-9^ | C | 9 | 0.0021 | 0 | 0 | 0 | 0 | 0.0012 | 20.4 | 12.24 | **‑**0.18 |
| Promoter/Enahncer | GFAP | 5:151095151:G:C  (rs77102485) | *TNIP1* | 0.0061 | C | 213 | 0.049 | 0.00058 | 0.065 | 0.21 | 0.14 | 0.047 | 5 | 1.34 | -0.02 |
| Promoter/Enhancer | GFAP | 7:100429736:T:C (rs537662968) | *ZCWPW1* | 2.01x10^-6^ | C | 13 | 0.0030 | 0 | 0 | 0 | 0 | 0.0035 | 0.503 | 11.54 | **‑**0.048 |

Ab42=amyloid β 42, AD=Alzheimer’s disease, AF=allele frequency, AFR=African, EAS=East Asian, GFAP=glial fibrillary acidic protein, MAC=minor allele count, MAF=minor allele frequency, MID=Middle Eastern, NFE=non-Finnish European, NfL=neurofilament light chain, pTau=tau phosphorylated at threonine 181, SAS=South Asian, tTau=total tau

**Table S11:** Estimates for the age effect and gene-by-age interaction effect for neurodegenerative biomarker genes

| Analysis | Neurodegenerative Biomarker | ID | Gene | GxE p | Minor Allele | MAC | MAF  (Analytic) | AF  (EAS) | AF  (AFR) | AF  (MID) | AF  (NFE) | AF  (SAS) | CADD  Phred | Main  Effect | Interaction  Effect |
| --- | --- | --- | --- | --- | --- | --- | --- | --- | --- | --- | --- | --- | --- | --- | --- |
| Missense/LoF | Ab42 | 19:44908822:C:T  (rs7412) | *APOE* | 0.00022 | T | 182 | 0.050 | 0.080 | 0.10 | 0.048 | 0.079 | 0.037 | 26 | 1.55 | -0.02 |

Ab42=amyloid β 42, AD=Alzheimer’s disease, AF=allele frequency, AFR=African, EAS=East Asian, GFAP=glial fibrillary acidic protein, MAC=minor allele count, MAF=minor allele frequency, MID=Middle Eastern, NFE=non-Finnish European, NfL=neurofilament light chain, pTau=tau phosphorylated at threonine 181, SAS=South Asian

***Supplemental Figures***

**Figure S1:** Correlation matrix of log_10_ z-score neurodegenerative biomarker measurements Winsorized to 5 standard deviations. The Pearson correlation coefficient was calculated for pairs of biomarkers: amyloid β 40 (Ab40), amyloid β 42 (Ab42), Ab42/Ab40, glial fibrillary acidic protein (GFAP), neurofilament light chain (NfL), tau phosphorylated at threonine 181 (pTau), and total tau (tTau).

** p<0.0001; * 0.0001≤p<0.05

**Figure S2:** Plot of missense/loss-of-function SNVs with MAC≥5 in *ECHDC3* gene for Ab40. Left Y-axis: -log10(p-value) from association between SNV and Ab40, adjusting for age, sex, and the first 10 principal components of genetic ancestry, and accounting for relatedness and plate as random effects; Right Y-axis: SNV recombination rate is averaged over all 1000 genomes GRCh38 l reference panels, lifted over from hg19, queried from the University of California, Santa Cruz; X-axis: chromosomal location and gene regions; LD information is calculated as the unphased r^2^ calculated on the LASI-DAD analytic sample; LD r^2^ color code indicates the degree of linkage between SNVs and the index (most strongly associated) SNV, rs35986488 (purple triangle). Dashed line is p=0.05. No annotation or MAF weighting was used to calculate p-values. Effect direction for SNVs with p<0.05 is indicated by triangle direction (up indicates positive effect, down indicates negative). The minor allele of the significant SNV (rs35986488) was associated with lower Ab40.

**Figure S3:** Plot of missense/loss-of-function SNVs with MAC≥5 in *EPHA1* gene for GFAP. Left Y-axis: -log10(p-value) from association between SNV and GFAP, adjusting for age, sex, and the first 10 principal components of genetic ancestry, and accounting for relatedness and plate as random effects; Right Y-axis: SNV recombination rate is averaged over all 1000 genomes GRCh38 l reference panels, lifted over from hg19, queried from the University of California, Santa Cruz; X-axis: chromosomal location and gene regions; LD information is calculated as the unphased r^2^ calculated on the LASI-DAD analytic sample; LD r^2^ color code indicates the degree of linkage between SNVs and the index (most strongly associated) SNV, rs141275729 (purple triangle). Dashed line is p=0.05. No annotation or MAF weighting was used to calculate p-values. Effect direction for SNVs with p<0.05 is indicated by triangle direction (up indicates positive effect, down indicates negative). The minor allele effect directions on GFAP were inconsistent, with the minor allele of the index SNV (rs141275729) associated with lower GFAP, which is linked to lower AD risk.

**Figure S4:** Plot of missense/loss-of-function SNVs with MAC≥5 in *CASS4* gene for pTau. Left Y-axis: -log10(p-value) from association between SNV and pTau, adjusting for age, sex, and the first 10 principal components of genetic ancestry, and accounting for relatedness and plate as random effects; Right Y-axis: SNV recombination rate is averaged over all 1000 genomes GRCh38 l reference panels, lifted over from hg19, queried from the University of California, Santa Cruz; X-axis: chromosomal location and gene regions; LD information is calculated as the unphased r^2^ calculated on the LASI-DAD analytic sample; LD r^2^ color code indicates the degree of linkage between SNVs and the index (most strongly associated) SNV, rs769966727 (purple triangle). Dashed line is p=0.05. No annotation or MAF weighting was used to calculate p-values. Effect direction for SNVs with p<0.05 is indicated by triangle direction (up indicates positive effect, down indicates negative). The minor allele of the significant SNV (rs769966727) was associated with lower pTau, which is linked to lower AD risk.

**Figure S5:** Plot of missense/loss-of-function SNVs with MAC≥5 in *ICA1L* gene for pTau. Left Y-axis: -log10(p-value) from association between SNV and pTau, adjusting for age, sex, and the first 10 principal components of genetic ancestry, and accounting for relatedness and plate as random effects; Right Y-axis: SNV recombination rate is averaged over all 1000 genomes GRCh38 reference panels, lifted over from hg19, queried from the University of California, Santa Cruz; X-axis: chromosomal location and gene regions; LD information is calculated as the unphased r^2^ calculated on the LASI-DAD analytic sample; LD r^2^ color code indicates the degree of linkage between SNVs and the index (most strongly associated) SNV, rs144482724 (purple triangle). Dashed line is p=0.05. No annotation or MAF weighting was used to calculate p-values. Effect direction for SNVs with p<0.05 is indicated by triangle direction (up indicates positive effect, down indicates negative). The minor allele of the significant SNV (rs144482724) was associated with higher pTau, which is linked to higher AD risk

**Figure S6:** Plot of missense/loss-of-function SNVs with MAC≥5 in *CLU* gene for Ab42/Ab40. Left Y-axis: -log10(p-value) from association between SNV and Ab42/Ab40, adjusting for age, sex, and the first 10 principal components of genetic ancestry, and accounting for relatedness and plate as random effects; Right Y-axis: SNV recombination rate is averaged over all 1000 genomes GRCh38 l reference panels, lifted over from hg19, queried from the University of California, Santa Cruz; X-axis: chromosomal location and gene regions; LD information is calculated as the unphased r^2^ calculated on the LASI-DAD analytic sample; LD r^2^ color code indicates the degree of linkage between SNVs and the index (most strongly associated) SNV, rs371557794 (purple triangle). Grey points indicate no LD information available. No annotation or MAF weighting was used to calculate p-values. Effect direction for SNVs with p<0.05 is indicated by triangle direction (up indicates positive effect, down indicates negative). The minor allele of the significant SNV (rs371557794) was associated with higher blood Ab42/Ab40, which is linked to lower AD risk.

**Figure S7:** Plot of brain-specific promoter/enhancer SNVs with MAC≥5 in *APOE* for Ab42/Ab40. Left Y-axis: -log10(p-value) of the association between the SNV and Ab42/Ab40, adjusting for age, sex, and the first 10 principal components of genetic ancestry, and accounting for relatedness and plate as random effects; Right Y-axis: SNV recombination rate averaged over all 1000 genomes GRCh38 reference panels, lifted over from hg19, queried from the University of California, Santa Cruz; X-axis: chromosomal location and gene regions. LD information is calculated as the unphased r^2^ from the LASI-DAD analytic sample. The LD r^2^ color code indicates the degree of linkage between the SNV and the index (most strongly associated) SNV, rs429358 (purple triangle). No annotation or MAF weighting was used to calculate the p-values. The effect direction for SNVs with p<0.05 is indicated by triangle direction (up indicates positive effect, down indicates negative). The minor allele effect directions on Ab42/Ab40 inconsistent, with the minor allele of the index SNV (rs429358, ε4) associated with lower Ab42/Ab40, which is linked to higher AD risk.

**Figure S8:** Plot of brain-specific promoter/enhancer SNVs with MAC≥5 in *APOC1* for Ab42/Ab40. Left Y-axis: -log10(p-value) of the association between the SNV and Ab42/Ab40, adjusting for age, sex, and the first 10 principal components of genetic ancestry, and accounting for relatedness and plate as random effects; Right Y-axis: SNV recombination rate averaged over all 1000 genomes GRCh38 reference panels, lifted over from hg19, queried from the University of California, Santa Cruz; X-axis: chromosomal location and gene regions. LD information is calculated as the unphased r^2^ from the LASI-DAD analytic sample. The LD r^2^ color code indicates the degree of linkage between the SNV and the index (most strongly associated) SNV, rs7256200 (purple triangle). No annotation or MAF weighting was used to calculate the p-values. The effect direction for SNVs with p<0.05 is indicated by triangle direction (up indicates positive effect, down indicates negative). The minor allele effect directions on Ab42/Ab40 inconsistent, with the minor allele of the index SNV (rs7256200) associated with lower Ab42/Ab40, which is linked to higher AD risk.

**Figure S9:** Plot of predicted standardized biomarkers by age and minor allele dosage of missense/loss-of-function variants with smallest interaction p-values in genes significantly interacting with age across the age range of LASI-DAD. Predicted values were estimated from a linear model adjusting for the variant, age, sex, and the first 10 PCs of global ancestry. The dashed line shows the mean predicted biomarker level. The bands represent 95% confidence intervals for the mean.

**Figure S10:** Plot of predicted standardized biomarkers by age and minor allele dosage of brain-specific promoter/enhancer variants with smallest interaction p-values in genes significantly interacting with age across the age range of LASI-DAD. Predicted values were estimated from a linear model adjusting for the variant, age, sex, and the first 10 PCs of global ancestry. The dashed line shows the mean predicted biomarker level. The bands represent 95% confidence intervals for the mean.
